# Asymptomatic Hyperuricemia Is Associated with Achilles Tendon Rupture through Disrupting the Normal Functions of Tendon Stem/Progenitor Cells

**DOI:** 10.1155/2022/6795573

**Published:** 2022-12-01

**Authors:** Jingjing Liang, Biao Chen, Yanmei Li, Daibang Nie, Changbin Lei, Qing Yang, Yiqin Zhou, Shenglong Li, Shuaizhi Chen, Bin Li, Lin Shu, Liaobin Chen, Wang Wang

**Affiliations:** ^1^Department of Immunology, College of Basic Medicine, Chongqing Medical University, Chongqing, China; ^2^Chongqing Key Laboratory of Basic and Translational Research of Tumor Immunology, Chongqing Medical University, Chongqing, China; ^3^Division of Joint Surgery and Sports Medicine, Department of Orthopedic Surgery, Zhongnan Hospital of Wuhan University, Wuhan, China; ^4^Department of Orthopedics, Affiliated Hospital of Xiangnan University, Chenzhou, China; ^5^Department of Orthopaedic Surgery, Tongji Hospital, Huazhong University of Science and Technology, China; ^6^Department of Orthopedics, Shanghai Changzheng Hospital, Naval Medical University, Shanghai, China

## Abstract

Hyperuricemia is a metabolic disorder that is essential to the development of inflammatory gout, with increasing prevalence over recent years. Emerging clinical findings has evidenced remarkable tendon damage in individuals with longstanding asymptomatic hyperuricemia, yet the impact of hyperuricemia on tendon homeostasis and associated repercussions is largely unknown. Here, we investigated whether asymptomatic hyperuricemia was associated with spontaneous ruptures in the Achilles tendon and the pathological effect of hyperuricemia on the tendon stem/progenitor cells (TSPCs). Significantly higher serum uric acid (SUA) levels were found in 648 closed Achilles tendon rupture (ATR) patients comparing to those in 12559 healthy volunteers. *In vitro* study demonstrated that uric acid (UA) dose dependently reduced rat Achilles TSPC viability, decreased the expressions of tendon collagens, and deformed their structural organization while significantly increased the transcript levels of matrix degradative enzymes and proinflammatory factors. Consistently, marked disruptions in Achilles tendon tissue structural and functional integrity were found in a rat model of hyperuricemia, together with enhanced immune cell infiltration. Transcriptome analysis revealed a significant elevation in genes involved in metabolic stress and tissue degeneration in TSPCs challenged by hyperuricemia. Specifically, reduced activity of the AKT-mTOR pathway with enhanced autophagic signaling was confirmed. Our findings indicate that asymptomatic hyperuricemia may be a predisposition of ATR by impeding the normal functions of TSPCs. This information may provide theoretical and experimental basis for exploring the early prevention and care of ATR.

## 1. Introduction

Gout is a type of inflammatory arthritis resulting from monosodium urate (MSU) crystal deposition in the joint tissues oversaturated with SUA [1–3]. SUA levels > 420 *μ*mol/L (7.0 mg/dL) in men and >360 *μ*mol/L (6.0 mg/dL) in women are diagnosed as hyperuricemia [4, 5]. To a less extent, crystallization of MSU inside the tendon tissues has been reported, which is preceded by hyperuricemia in the interstitial fluids [6–9]. It has been evidenced that direct interaction of MSU crystals with tenocytes could reduce cell survival and functions, which correlated with tendon injuries in patients with advanced gout [6]. Longstanding hyperuricemia challenges the histological and functional properties of tendon because of their potential to induce regional inflammation and metabolic distress [10–12]. Remarkably, subclinical tendon damage is presented in asymptomatic hyperuricemia [9, 13]. Hence, a tentative relationship between hyperuricemia and the vulnerability of tendon tissue has long been questioned, yet with very few collective reports available [9, 12–14].

Achilles tendon rupture (ATR) is the most common tendon injury in the lower extremity, with rising incidents in individuals engaged in regular physical exercise or recreational sports [15, 16]. The direct cause of ATR is usually a sudden stretch of the Achilles tendon (AT), while degeneration in AT prior to the rupture has been evidenced in over 80% of ATR cases [15–18]. In normal condition, the biomechanical adaptation of AT to extrinsic and intrinsic influences relies on proper functions of tendon stem/progenitor cells (TSPCs) [17, 19]. This small subset of cells within tendon tissue possesses clonogenic capacity and plays a vital role in the maintenance of tendon homeostasis [17–19]. Multiple factors, including overuse, aging, metabolic disorders, and the use of fluoroquinolones and corticosteroids, have been shown to induce cellular deficits in TSPCs and are closely associated with the pathological process of ATR [15, 20–22]. Scattered evidences have linked hyperuricemia with ATR, in accordance with the assumption that the functional integrity of tendon tissue may be compromised by the elevated levels of SUA [6, 14]. However, the effect of hyperuricemia on TSPCs and the associated molecular mechanism is still unclear.

The aim of this study was to investigate the relationship between hyperuricemia and ATR. We assessed the correlation of SUA levels to ATR incidence in a substantial population of participants with or without spontaneous rupture of AT and explored the cellular and molecular alterations on TSPCs and tendon homeostasis brought by UA *in vitro* and *in vivo*.

## 2. Materials and Methods

### 2.1. Study Participants and Data Collection

A total of 648 patients with closed ATR were enrolled from January 2010 to August 2021. At the same time, 12559 volunteers who were engaged in physical examination in the Physical Examination Center of Zhongnan Hospital were recruited as healthy controls. The study was conducted according to the Declaration of Helsinki. Ethical approval for the study was obtained from the Ethics Committee of Zhongnan Hospital, Wuhan University (Wuhan, China), and informed consent was obtained from all participants. Exclusion criteria were age < 18 years, current or previous use of urate-lowering agents and nonsteroidal anti-inflammatory drugs or corticosteroids, and malignancy or other concomitant rheumatic inflammatory conditions.

### 2.2. Preparation of Soluble UA

Sodium hydroxide solution (NaOH) was used as a solvent prepared by dissolving 400 milligrams (mg) of NaOH in 10 milliliters (ml) of double distilled water (ddH_2_O); then, 336 mg UA (Sigma-Aldrich, MO, USA) was added to the solvent and dissolved at 65°C. Different concentrations of soluble UA were diluted with high glucose DMEM (Gibco, MA, USA) with 10% fetal bovine serum (FBS) (ExCell Biology, Jiangsu, China), and the media was placed in cell incubator 1 hour (h) prior to treatment to allow for pH adjustment.

### 2.3. Animals and Cells

TSPCs were isolated from AT tissue of Sprague-Dawley rats (male = 6, female = 6; 8 weeks) as previously described [23]. The protocol for the use of rats was approved by the Institutional Animal Care and Use Committee (IACUC) of Chongqing Medical University. For the hyperuricemia rat model, 8-week-old Sprague-Dawley rats were assigned to the hyperuricemia group (*n* = 12) and the control group (*n* = 12). Rats in the hyperuricemia group were intragastrically administered with adenine (0.1 grams/kilogram, Macklin, Shanghai, China) and potassium oxonate (1.5 grams/kilogram, Macklin) dissolved in 0.5% sodium carboxymethylcellulose (Macklin) once per day for 6 successive weeks. On the same days, rats in the control group were intragastrically administered with equal volumes of 0.5% sodium carboxymethylcellulose only.

### 2.4. Proliferation, Cell Cycle, and Apoptosis Assays

Cells cultured in 96-well plates (1500 cells/well) were treated with different concentrations of UA for various times before subjected to Cell Counting Kit-8 (CCK-8, Bioss, MA, USA) assay to determine cell viability. For cell cycle analysis, cells were fixed in 75% ethanol. After washing with PBS, cells were stained by propidium iodide (PI, MedChemExpress, NJ, USA) in staining solution supplemented with 0.2% Triton X-100 (Solarbio, Beijing, China) and RNase A (Solarbio). Cell cycle was assessed using a flow cytometer (BD FACSCelesta, OA, USA). For cell death detection, cell samples were incubated with 0.5 *μ*L APC-Annexin V and 7-AAD (BioLegend, CA, USA). The number of apoptotic cells in 1 × 10^5^ cells was counted by a flow cytometer (BD FACSCelesta).

### 2.5. Histology

The AT specimens were collected from the animal immediately after sacrifice (*n* = 5 from the control group; *n* = 6 from the hyperuricemia group). The specimens were fixed in 4% paraformaldehyde (NCM Biotech, Suzhou, China) for 72 h and then embedded in paraffin. Sections that were 4 micrometers thick were stained with hematoxylin and eosin (H&E, Solarbio) and imaged by a slide scanner for digital pathology (Leica DM300, AG, Germany).

### 2.6. Immunohistochemistry and Immunofluorescence Analysis

The multidifferentiation potential of the TSPCs treated with various concentrations of UA was tested by oil red O staining for adipogenesis, by safranin O staining for chondrogenesis, and by alizarin red S staining for osteogenesis, as previously described [23]. Masson's trichrome (MT) staining (Solarbio) and Sirius red staining (Solarbio) were used to detect collagen fiber deposition. For immunofluorescence, tendon sections were sealed with 5% BSA containing 0.1% Triton-X, then incubated with primary antibody (CD3, 1 : 200, Proteintech, IL, USA; CD68, 1 : 200, Affinity, OH, USA), washed, and incubated with secondary antibodies (Antirabbit IgG (H+L), F(ab′)_2_ Fragment Alexa Fluor® 488 Conjugate, 1 : 500, Cell Signaling Technology, MA, USA). The stained cells were observed and imaged (Leica DMi8, AG, Germany).

### 2.7. RNA Extraction and Quantification

Total RNA was extracted with the RNA Extraction kit (Beyotime, Shanghai, China), and the concentrations were measured with the NanoDrop 2000 (Thermo Fisher Scientific, MA, USA). The cDNA was synthesized using the RevertAid Master Mix (Thermo Fisher Scientific). Real-time quantitative polymerase chain reaction (RT-qPCR; Platinum® qPCR SuperMix-UDG, Thermo Fisher Scientific) was performed by a CFX96 Touch qPCR System (Bio-Rad, CA, USA) as previously described [23], using specific sets of primers/probes for genes of interest (Supplementary Table 1). Gene expressions were assessed in relation to the levels of *β*-actin.

### 2.8. Western Blot Analysis

Cells cultured in a 10-centimeter dish (5 × 10^4^ cells/plate) were treated with different concentrations of UA for 48 h; then, cell lysates were processed for Western blot analysis. Primary antibodies were used in 1 : 1000 dilutions for anti-phospho-AKT (AF0016, Affinity), anti-AKT (AF6261, Affinity), anti-phospho-mTOR (5536 s, Cell Signaling Technology), anti-mTOR (2983 s, Cell Signaling Technology), anti-phospho-p70S6K (AF3228, Affinity), anti-p70S6K (AF6226, Affinity), and anti-phospho-4E-BP1 (2855 s, Cell Signaling Technology). Additional primary antibodies used were anti-4E-BP1 (1 : 500, 60246-1-Ig, Proteintech), anti-p62 (1 : 750, AP6006, Bioworld, MN, USA), anti-LC3A/B (1 : 600, 14600-1-AP, Proteintech), and anti-*β*-actin (1 : 20000, AF7018, Affinity).

### 2.9. Biomechanical Testing

Mice from AT samples were harvested from the animals (*n* = 7 from the control group; *n* = 6 from the hyperuricemia group) immediately after sacrifice and frozen at -80°C until testing. The specimens were thawed overnight at room temperature. Biomechanical testing was performed using a materials testing machine (805, Instron Co., MA, USA). The tendon tissue was fixed between the U-shaped clamps to ensure that the pulling force was parallel to the axis of the tissue, with a displacement rate of 10 millimeters/minute. The ultimate failure load and stiffness were determined from the load-displacement curve.

### 2.10. Statistical Analysis

Unless otherwise indicated, data are expressed as the mean ± SEM. Statistical significance was determined by Student's *t*-test or by Mann–Whitney *U* test for comparisons of continuous variables between 2 groups or by one-way or two-way repeated-measures analysis of variance (ANOVA) when group numbers are more than 2. *P* values less than 0.05 were considered significant. The correlation between parameters was evaluated with Pearson's correlation coefficient.

## 3. Results

### 3.1. Relationship of SUA Levels to ATR

**Figure 1:**
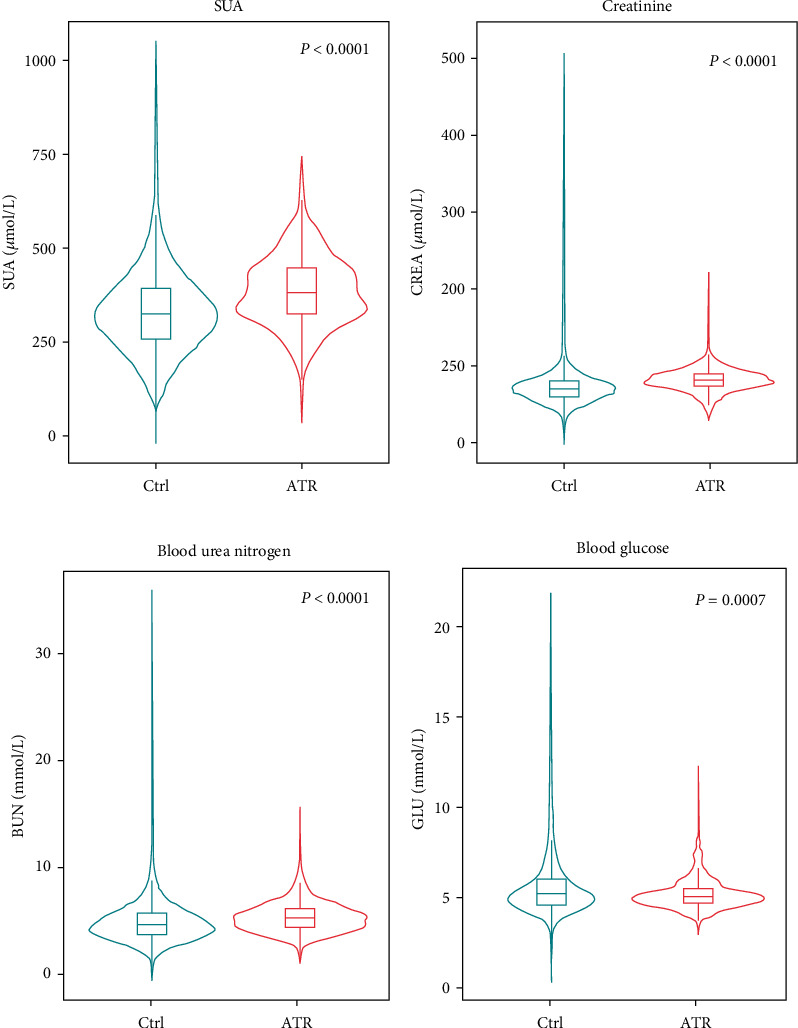
Comparison analyses between the ATR group and the control subjects. The levels of SUA (*P* < 0.0001) (a), creatinine (*P* < 0.0001) (b), blood urea nitrogen (*P* < 0.0001) (c), and blood glucose (*P* = 0.0007) (d) exhibited significant differences in ATR patients (*n* = 648) and the control group of volunteers (*n* = 12559).

Samples had been collected from 648 ATR patients over a course of 12 years, of which, 91.5% were male (Table 1). Cross-sectional analysis revealed that SUA levels were significantly higher in ATR patients than in the gender-matched control group of 12559 volunteers (Figure 1(a), Table 1). The prevalence of hyperuricemia was 35.96% in ATR patients and 18.03% in the control group (Table 1), with an odds ratio of 2.612 for hyperuricemia in the occurrence of ATR in the total population (95% confidence interval: 2.215-3.080, *P* < 0.01). Consistently, ATR patients had greater levels of both creatinine and blood urea nitrogen than the control group (Figures 1(b) and 1(c), Table 1). The blood glucose was significantly lower in ATR patients (Figure 1(d), Table 1). There was a 5-year difference in the average age between the participants of these 2 groups. The ages of the ATR patients, ranged from 18 to 60 years old as matched to the control subjects, exhibited a positive correlation with the incidence rate of ATR in our cohort (Supplementary Figure 1). The systolic and diastolic blood pressure levels were comparable between ATR patients and the control subjects (Table 1). Besides, no significant differences were found in the levels of triglyceride and total cholesterol from different groups (Table 1).

### 3.2. UA Inhibits Cell Viability and Promotes Nontenocytic Differentiation of Rat TSPCs

**Figure 2:**
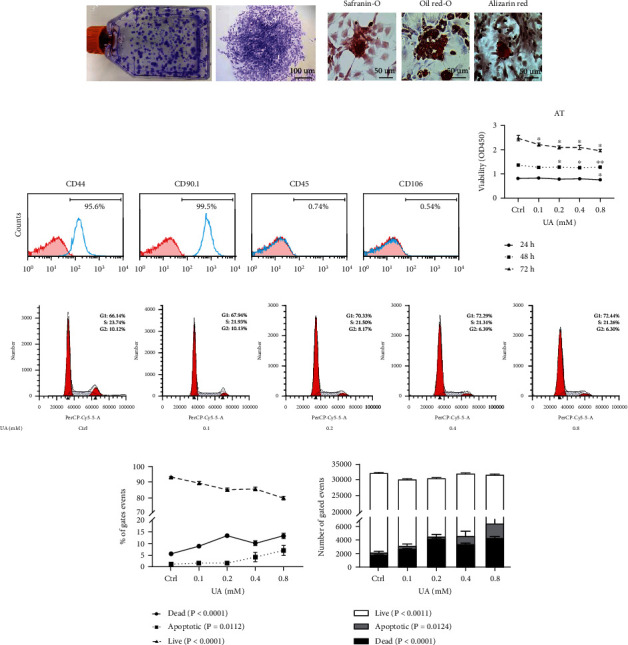
Effects of UA treatment on TSPC viability and survival. Characteristics of primary cells extracted from SD rat tendon were determined in (a)–(c). (a) Primary cell colonies stained with crystal violet at 12 days. (b) Primary cells cultured under differentiation conditions for 21 days, followed by safranin O, oil red O, and alizarin red S staining to detect chondrogenic, adipogenic, and osteogenic differentiation, respectively. (c) Flow cytometry analysis of the expression of cell surface markers related to stem cells (CD44 and CD90.1), leukocyte (CD45), and endothelial cell (CD106). (d) CCK8 assays were used to assess TSPC proliferation over a course of 72 h, with the addition of PBS or UA (0.1, 0.2, 0.4, and 0.8 mM). (e) Representative images show cell cycle distribution with flow cytometric analysis with UA treatment after 48 h. Changes in the percentile (f) and numbers (g) of live, apoptotic and dead TSPCs were assessed over 48 h with UA treatment. A control treated with PBS was included in all experiments. Results are presented as the mean ± SEM from three independent experiments, using TSPC isolates from 3 individual rats. Data were analyzed using ANOVA. ^∗^*P* < 0.05; ^∗∗^*P* < 0.01 versus PBS-treated control at the relevant time point.

To characterize isolated tendon-derived cells, single-cell suspensions were generated from 8-week-old SD rats. Large colonies were readily observed after 8 days (Figure 2(a)). When subjected to differentiation media, these cells exhibited profound capabilities to form different cell lineages. Obvious lipid droplets were observed by oil red O staining after adipogenic induction (Figure 2(b)). Also, clump formation was readily observed by safranin O assay postchondrogenic induction, and red calcium deposition could be visualized by alizarin red staining with cells exposed to osteogenic inducing media for 14 days (Figure 2(b)). Flow cytometry analysis was performed to examine surface antigens on these cells. We found that over 95% of the cell population from the primary generation expressed mesenchymal stem cell markers CD44 and CD90.1, and more than 99% cells were negative for leukocyte marker CD45 and endothelial cell marker CD106 (Figure 2(c)). These results indicate that these cells isolated from rat tendon exhibit stem cell properties and are practical resources for the study of TSPCs.

In order to understand the correlation of elevated levels of SUA to ATR, we first investigated the effect of UA on TSPCs. Soluble UA inhibited the proliferation of TSPCs isolated from rat AT in a dose-dependent manner, as tested by the CCK8 assay (Figure 2(d)). In parallel, cell cycle analysis identified a dose-dependent decrease in the number of TSPCs in the G2/S phase when treated with different levels of UA (Figure 2(e)). Flow cytometry analysis showed that the reduced viability of TSPCs was accompanied by a significant increase of cell death with UA treatment, even at low concentrations (Figures 2(f) and 2(g); Supplementary Figure 2). These events occurred without profound evidence of apoptosis (Figures 2(f) and 2(g)).

**Figure 3:**
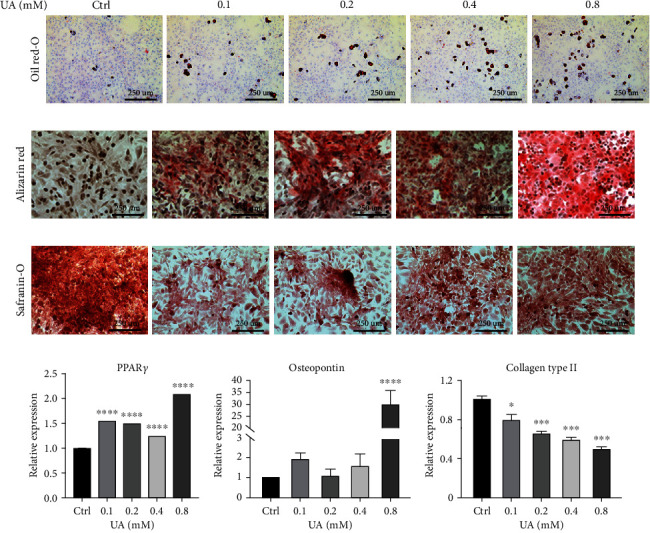
Effects of UA on the nontenocytic differentiation of TSPCs. TSPCs were subjected to three types of differentiation media with the addition of PBS or various concentrations of UA for 21 days. (a–c) Subsequent to the differentiation course, oil red O staining (a), alizarin red staining (b), and safranin O staining (c) were performed to assess changes in adipogenesis, osteogenesis, and chondrogenesis of rat TSPCs, respectively. Representative images of each group show morphological alterations and visible changes in staining intensity (*n* = 3 each). Bars = 250 *μ*m. (d–f) Real-time qPCR analysis was applied to determine expression of marker genes in TSPC postcomplete differentiation. PPAR*γ* was used as the marker of adipogenic differentiation (d), with osteopontin for osteogenic (e), and collagen type 2 for chondrogenic differentiation (f). Values are the mean ± SEM from three independent experiments. Data were analyzed using two-way ANOVA. ^∗^*P* < 0.05; ^∗∗∗^*P* < 0.001; ^∗∗∗∗^*P* < 0.0001 versus PBS-treated control within each group.

To further test whether UA affected the regular functions of TSPCs, we examined the adipogenic, osteogenic, and chondrogenic capacity of TSPCs under the treatment of UA. After 21 days of differentiation culture, fat vacuoles were readily observed by oil red O staining, and UA treated TSPCs exhibited higher degree of adipogenic potentials than the control group treated with PBS (Figure 3(a), Supplementary Figure 3a). Similarly, osteoblast-like changes with irregular cuboidal morphology were detected in TSPCs treated with UA (Figure 3(b)). Alizarin red S staining showed that the formation of calcium nodules was positively correlated with the concentration of UA treatment (Figure 3(b), Supplementary Figure 3b). In contrast, safranin O staining demonstrated an evidently higher level of glycosaminoglycan (GAG) deposition produced by the control TSPCs than those UA treated groups, indicating that UA inhibited the process of TSPC chondrogenic differentiation (Figure 3(c), Supplementary Figure 3c). In parallel, gene expression analysis confirmed that PPAR*γ* and osteopontin, as the marker of adipogenic and osteogenic differentiation, respectively, were both upregulated by UA treatment than the control, especially with the highest dose of 0.8 mM (Figures 3(d) and 3(e)). Meanwhile, chondrogenic marker collagen type 2 was significantly downregulated in TSPCs exposed to UA treatment relative to the PBS control (Figure 3(f)). These results indicated that elevated levels of UA could impede the normal functions of TSPCs, by reducing the cell viability, and promote their adipogenic and osteogenic differentiation while inhibiting the formation of chondrogenic lineages.

### 3.3. Hyperuricemia Induces Pathological and Mechanical Changes in Rat AT

**Figure 4:**
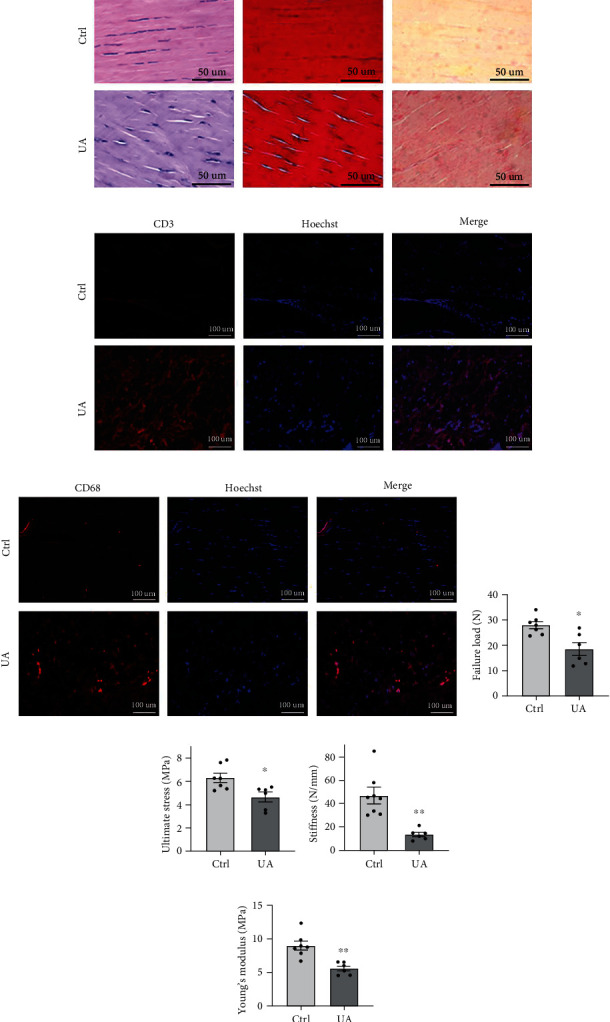
Characterization of the AT tissue from the experimental rat model of hyperuricemia. (a–c) Representative sections of AT from rats intragastrically administered with 0.5% sodium carboxymethylcellulose (*n* = 5) or UA (*n* = 6). Tendon tissues showed remarkable differences in the morphology by hematoxylin and eosin staining ((a), left) and in the collagen expression by Masson's trichrome (MT) staining ((a), middle) and Sirius red staining ((a), right). Bars = 50 *μ*m. (b, c) Representative sections were stained for CD3 (b) and CD68 (c) in red, and nuclei were stained with Hoechst 33258 (blue). Bars = 100 *μ*m. (d–g) Comparison of mechanical testing results of the rat AT tissue postinduction of UA (*n* = 6) or the vehicle control (*n* = 7) in the failure load (d), the ultimate stress level (e), the stiffness (f), and the levels of Young's modulus (g). Values are the mean ± SEM of average data from 2 independent experiments. ^∗^*P* < 0.05; ^∗∗^*P* < 0.01 versus vehicle alone.

To investigate the effects of hyperuricemia on the properties of the Achilles tendon, histopathological analysis was performed with the AT tissue collected from a known hyperuricemia rat model [24]. H&E staining showed that the AT cells in the control group were fusiform and arranged in a parallel and uniform manner, and the collagen fibers were smooth and orderly arranged (Figure 4(a)). AT cells of the hyperuricemia group appeared oval in shape, and the cell numbers were notably reduced (Figure 4(a)). Also, the matrix around AT cells from the hyperuricemia animals was basophilic with obvious collagen disintegration and disorientation (Figure 4(a)). Meanwhile, as shown by Masson's trichrome (MT) staining and Sirius red staining, the interstitial structure was disordered in the hyperuricemia group, and the fibrous tissue was presented with extensive hyperplasia compared with the control group (Figure 4(a)). These highly increased levels of collagen deposition and disorganization indicated that hyperuricemia could induce structural alterations in the AT tissue.

Given the established association of gout with systematic and regional inflammation, we assessed the infiltration of inflammatory cells in rat AT tissues in the presence of hyperuricemia. Immunofluorescence staining for CD3 and CD68 was performed to assess the infiltration of T cells and macrophages, respectively (Figures 4(b) and 4(c), Supplementary Figures 4a and 4b). Within the AT tissue from rats with hyperuricemia, evidently increased numbers of T cells (Figure 4(b)) and macrophages (Figure 4(c)) were both detected, in comparison with the control group. These results confirmed that hyperuricemia could induce active infiltration of immune cells in the AT tissue, which was consistent with previous findings [6, 7]. We also confirmed whether UA directly affect the mRNA expressions of inflammatory cytokines and ECM-related factors in the TSPCs. Real-time PCR results showed that genes of proinflammatory cytokines, such as interleukin (IL)-1*β*, IL-6, and tumor necrosis factor- (TNF-) *α*, and degradative enzymes, such as matrix metalloprotease (MMP) 2, MMP3, and MMP13 (Supplementary Figures 4c and 4d), were both significantly upregulated after 48 hours of culture with UA than the PBS control. These results demonstrated that hyperuricemia could bring histological disturbance in the AT tissue, with enhanced immune cell infiltration, which was in consistence with elevated proinflammatory responses and catabolic gene expressions in TSPCs.

In order to investigate whether the observed pathological alterations in AT brought by hyperuricemia were associated with an overall impact on AT mechanical properties, we performed a series of mechanical tests with AT samples in a biomechanical loading system. The failure load and the average ultimate force in AT from rats with hyperuricemia were both significantly lower than the native AT (Figures 4(d) and 4(e)). In accordance, significant differences were shown in the stiffness and Young's modulus of AT between the two groups (Figures 4(f) and 4(g)). Altogether, these results demonstrated that hyperuricemia could reduce the mechanical properties of AT, which implied a potential contributing role of hyperuricemia to the frequently observed pathology in the lower limbs in gout patients [7, 25].

### 3.4. UA Affects the Metabolic Process in Rat TSPCs by Downregulating mTOR Signaling

**Figure 5:**
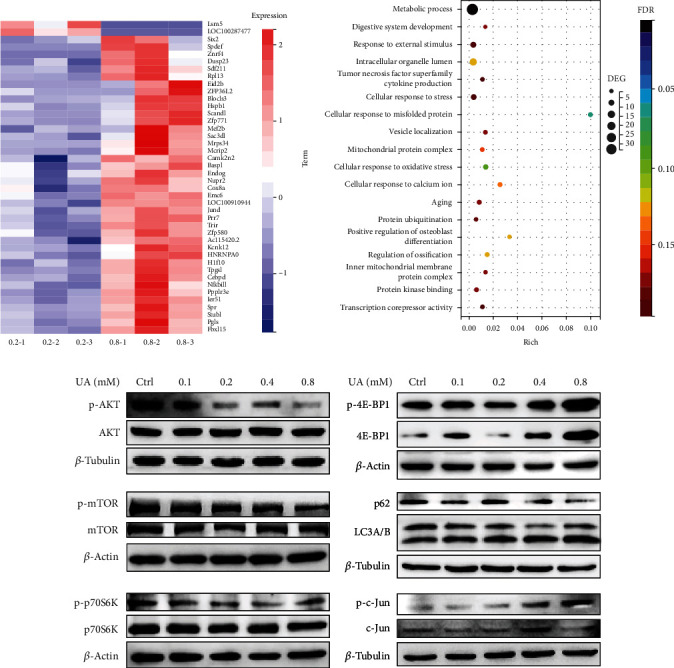
Analysis of the transcriptome and protein expressions in TSPCs treated with different concentrations of UA. (a) Heat map visualization of DEG between TSPCs exposed to UA at the physiological level (0.2 mM) and the level corresponding to hyperuricemia (0.8 mM) for 48 h. (b) GO enrichment of gene sets from TSPCs impacted by hyperuricemia. (c) Cell lysates of TSPCs treated with PBS or various doses of UA for 48 h were tested for the AKT-mTOR pathway, the autophagy markers, and c-Jun activation by Western blot analysis. Results are representative of at least 3 independent experiments.

To assess detailed information on the genetic profile of TSPCs affected by UA and to identify potential signaling pathways involved, transcriptome study was performed using bulk mRNA sequencing with RNA samples of TSPCs treated with PBS and two doses of UA for 48 hours. Analysis of the differential expressed genes (DEGs) revealed a similar transcriptome profile from the PBS-treated control TSPCs and the group treated with physiological level of UA (0.2 mM; Supplementary Figure 5). In comparison to the 0.2 mM UA-treated group, 40 genes were upregulated and 2 genes were downregulated in TSPCs treated with the UA level representing hyperuricemia (0.8 mM), as visualized in the heat map (Figure 5(a)). Gene Ontology (GO) enrichment analysis revealed that hyperuricemia affected TSPCs by influencing the expressions of genes majorly involved in the regulation of metabolic process, stress response, and degeneration (Figure 5(b)). In a subsequent evaluation of key molecular pathways associated with the cellular response of TSPCs to UA, we identified that the AKT/mTOR/autophagy signaling activity was profoundly impacted by UA treatment, in a dose-dependent manner (Figure 5(c), Supplementary Figure 6). Specifically, a treatment with different concentrations of UA for 48 hours markedly reduced the levels of AKT and mTOR phosphorylation, as shown by Western blot analysis (Figure 5(c), Supplementary Figure 6). These were associated with reduced expression of phosphoproteins of P70 S6 Kinase (P70 S6K) and increased expression of p-4E-BP1, and with decreased levels of LC3 A/B ratio and p62 expression (Figure 5(c), Supplementary Figure 6). Notably, addition of 0.8 mM UA in the TSPC culture remarkably increased the expression of phospho-c-Jun, which was in functional consistence with two of the upregulated genes identified above from the mRNA profiling, prr7, and jund (Figure 5(c)). Collectively, these findings reveal a positive correlation between hyperuricemia and ATR. High levels of UA not only disrupt the structural and functional integrity of AT but also directly impede tendon homeostasis by deregulating the normal functions of TSPCs.

## 4. Discussion

With the gradual changes in diet, from the traditional carbohydrate and vegetable-based pattern to the current one dominated by meat, dairy products, and other foods rich in purines, the number of reported cases of hyperuricemia and gout is rising [1]. In our healthy control group, the prevalence of hyperuricemia was 18.03%, which was in accordance with Liu's meta-analysis report in mainland China [26]. In the ATR group, the prevalence of hyperuricemia was 35.96%. This remarkable difference reinforced the clinical study reported by Dodds and Burry [14]. The relationship between hyperuricemia and inflammatory diseases has drawn broad attentions over the last decades. However, the precise role of high level SUA in tendon is still less understood. There is a general consensus that hyperuricemia, even asymptomatic, could promote a proinflammatory reaction within tendon tissue that contributes to the onset and progression of tendon abnormalities [7, 27]. Hence, it would be important to determine whether high level SUA contributed to the degenerative progression of AT and created a predisposition of ATR, which was the aim of this study.

Clinical findings between 648 closed ATR patients and 12559 gender-matched volunteers demonstrated a significant elevation in the SUA levels associated with tendon rupture, together with enhanced levels of creatinine and blood urea nitrogen. With this in mind, we addressed the effects of UA in TSPCs isolated from rat AT, which are the essential cell population in the maintenance of tendon homeostasis [19, 28]. Also, we assessed the impact of hyperuricemia on AT tissue structural and functional integrity. Furthermore, we characterized the molecular mechanisms for the disturbance on TSPCs when exposed to elevated concentrations of UA. Findings of this study linked hyperuricemia to ATR with clinical evidences and suggested that high level UA rendered the integrity of AT by directly reducing the viability of TSPCs and impairing their tenocytic differentiation, probably through the introduction of metabolic stress.

Advancement in crystal biology directly evidenced the involvement of MSU in the pathophysiological progression leading to tendinopathy [1, 29]. The harsh disturbance in the microenvironment within tendon tissues that was induced by the crystal depositions of UA often involved the proinflammatory milieu and caused irreversible changes in extracellular matrix composition [9, 30]. This concept was also confirmed by a reported causal relationship between MSU and tenocyte damage [6]. Importantly, our study demonstrated that prior to the formation of urate crystal deposition, elevation in UA levels could readily reduce the availability of the TSPC population within AT and impede their normal functions. This suggested that high level of UA could start to potentiate the tendinous pathophysiological progression, which closely resembled Achilles tendinosis, early during the asymptomatic stages of hyperuricemia. In accordance, the observed increase of CD3^+^ and CD68^+^ cells within the AT of hyperuricemia rats suggested that active sterile inflammation could be triggered and might collectively contribute to the degenerative changes induced by high level UA, which was commonly found within ruptured tendons [16, 17]. Whether there was a causal relationship between malfunctioned TSPCs and the inflammatory infiltration within the AT tissue could not be answered at this point. Nevertheless, elevation in UA levels could negatively affect tendon vulnerability and could be considered as a predisposing factor for ATR.

As reported, deteriorating integrity of tissue architecture rendered tendon to be more prone to tear and rupture [9, 12]. In our study, the mRNA expression of catabolic MMPs was shown to be dose-dependently promoted in TSPCs by exposure of UA, indicating an active response leading to the degradation of tendon matrix. Although an opposite reaction was reported in tenocytes stimulated with MSU crystal [20], our histological analysis of AT samples from rats with hyperuricemia showed evident reduction of homeostatic collagen tension relative to the normal control, which was accompanied by significantly impaired mechanical properties. These findings were consistent with observations in the tendons of gout patients [6, 8, 29]. Corroborating these data, hyperuricemia might trigger unique effects on TSPCs, probably distinct from those on tenocytes, by restricting the normal anabolic functions to replenish challenged tendon tissues as well as promoting the catabolic response of extracellular matrix. These events might collectively lead to disorganized tendon architecture. Also, recent studies have demonstrated the functional potential of TSPCs to differentiate into fibrocartilage-like cells [31] to constitute the fibrocartilage transition zone, which played an essential role in the bone-tendon junction [32, 33]. In light of our results and these findings, the role of hyperuricemia in TSPCs is of particular importance for ATR patients, particularly during the postrupture recovery process [17, 18]. It is reasonable to speculate that high levels of UA, in conjunction with the already low cellularity and vascularity within the injured AT, may raise the challenges of tendon replenishment and reconstruction from cells with stemness, which will be analyzed in the future study.

Accumulating evidences from genetic and epigenetic studies with clinical samples has pointed out that majority of gout candidate genes are directly associated to MSU crystals, including those influencing the crystal formation, the immune recognition of crystal deposits, and corresponding cellular responses to the proinflammatory milieu [34, 35]. In the exploration of hyperuricemia-associated transcriptome profiles in TSPCs, we identified pathway enrichment in metabolic process, responses to stress, and degeneration, all of which were fundamental in the fast response of TSPCs [19, 28]. Parallel analysis at the molecular level confirmed that the AKT-mTOR signaling activity was indeed suppressed by high level UA in the TSPCs. It is important because this pathway plays an essential role in sensing cellular stress and metabolic abnormalities, and disturbance of signaling activities downstream of mTOR in TSPCs is concomitant with mechanical stimulation and the regional hypertrophic response [23, 36]. Therefore, our findings provided a possible molecular explanation of how hyperuricemia affects the mechanical functions of AT. Of note, prr7, which was shown to enhance the stability of c-Jun [37], and jund, another component of the c-Jun subfamily within the AP-1 complex [38], were both found to be transcriptionally upregulated by hyperuricemia. In consistence, a dose-dependent increase in the phosphorylation of c-Jun was detected in TSPCs treated with UA. These results implied that UA exposure in TSPCs might lead to an activation of the AP-1 transcription factor complex, which had been evidenced to promote MMP production and mediate tissue degeneration [38, 39]. Together, our findings highlighted changes in metabolic programming of TSPCs and in ECM remodeling, corroborated the cellular response of TSPCs to UA treatment and the histological observations in rat AT with hyperuricemia. Detailed confirmations are still needed to determine the involvement of these molecules and their functional association with the overall effects of hyperuricemia on the tendon tissues.

There are other limitations with this study. The number of subjects studied here was relatively small, and further analysis of clinical data with a larger sample size from multiple centers would bring seminal value to understand the correlation between SUA abnormalities and ATR. Another limitation is the lack of comprehensive adjustment for the influence of hyperuricemia-associated comorbidities on tendon dysfunction and their associations with ATR. With the identified modulatory role of hyperuricemia on TSPC metabolic programming, we cannot exclude the possibility whether pertinent factors like dietary choices, obesity, type 2 diabetes, or previous use of medications may have contributed to the degeneration within tendon tissue independent of an elevated level of SUA.

In summary, we identified a positive correlation of hyperuricemia to ATR, and we demonstrated that elevated levels of UA might directly reduce the viability of TSPCs and impede their normal functions in regulating tendon homeostasis. Also, we evidenced that hyperuricemia disrupted the structural and mechanical integrity of AT. Further, we elucidated novel genes and pathways involved in hyperuricemia-induced deregulation of TSPCs.

## 5. Conclusion

Findings of this study may help to advance the understanding for the regulatory mechanisms with potential targets for hyperuricemia-associated tendon pathological process and highlight asymptomatic hyperuricemia as a concerning factor in the care for ATR patients.

## Figures and Tables

**Table 1:** Demographic and clinical features in subjects with and without ATR^1^.

Characteristics	ATR group (*n* = 648)	Non-ATR group (*n* = 12559)	*P* value
Gender (male/female)^2^	595/53	11490/1069	—
Age (years)	39.5 ± 10.6	34.5 ± 9.4	<0.0001
Range (years)	18-84	18-60	—
Systolic blood pressure (mmHg)	126.3 ± 13.0	126.2 ± 11.0	0.836
Diastolic blood pressure (mmHg)	79.3 ± 10.8	80.2 ± 11.5	0.367
SUA (*μ*mol/L)	385.7 ± 93.8	331.0 ± 108.0	<0.0001
Prevalence of hyperuricemia	35.96%	18.03%	—
Creatinine (mmol/L)	80.6 ± 14.8	85.4 ± 109.0	<0.0001
Urea nitrogen (mmol/L)	5.3 ± 1.4	4.9 ± 1.8	<0.0001
Blood sugar (mmol/L)	5.3 ± 1.0	5.7 ± 2.0	0.0007
Triglyceride (mmol/L)	1.6 ± 1.1	1.6 ± 1.2	0.95
Total cholesterol (mmol/L)	4.2 ± 0.8	4.2 ± 0.9	0.561

^1^Values are the mean ± SD. SUA: serum uric acid. *P* values between two groups are calculated by Mann–Whitney *U* test for comparisons of continuous variables. Results were the average measurements of two independent blood tests taken 7 days apart. ^2^The number of corresponding participants.

## Data Availability

The data used to support the findings of this study are available from the corresponding authors upon request.

## Supplementary Materials

Supplementary Materials Supplementary Table 1: primer sequences used in real-time PCR. Supplementary Figure 1: Pearson's correlations of age and ATR incidence (*P* < 0.0001). The incidences of ATR were calculated as the percentile of ATR patient numbers to the total number of participants of the same age. Age range was 18-60 years old, as matched to the control group. Supplementary Figure 2: flow cytometric analysis of annexin V-PI staining results for TSPCs treated with different doses of UA. Supplementary Figure 3: quantification for the oil red O staining (a), alizarin red staining (b), and safranin O staining (c) images in Figure 3 of TSPC differentiation potential when treated with different doses of UA (*n* = 3). Data were the normalized ratio of staining intensity of each treatment group over that of the PBS control. Values are the mean ± SD staining intensity of representative images of three independent experiments. ^∗^*P* < 0.05; ^∗∗^*P* < 0.01; ^∗∗∗∗^*P* < 0.0001 versus PBS control. Supplementary Figure 4: quantification of CD3 (a) and CD68 (b) immunofluorescent staining results in Figures 4(b) and 4(c), respectively (*n* = 3). Values are the mean ± SD staining intensity of representative images of three independent experiments. Real-time qPCR results of proinflammatory markers (c) and degradative enzymes (d) in TSPCs treated with different concentrations of UA or PBS (*n* = 3 each). Values show the mean ± SEM. ^∗^*P* < 0.05; ^∗∗^*P* < 0.01; ^∗∗∗^*P* < 0.001; ^∗∗∗∗^*P* < 0.0001 versus PBS-treated control within each group analyzed using two-way ANOVA. Results are from two independent experiments. Supplementary Figure 5: Venn diagrams illustrate the sharing of DEG between TSPCs treated with control (PBS) or different concentrations of UA. Ctrl: TSPCs treated with PBS; T02: TSPCs treated with 0.2 mM of UA; T08: TSPCs treated with 0.8 mM UA. The numbers (13, 29, 27, and 3) indicate the DEG between two groups. Supplementary Figure 6: quantification for Western blot analysis images in Figure 5(c) of TSPCs treated with different doses of UA (*n* = 3). Data were the normalized ratio of indicated protein expression over that of the corresponding loading control. Values are the mean ± SEM from three independent experiments. ^∗^*P* < 0.05; ^∗∗^*P* < 0.01; ^∗∗∗^*P* < 0.001; ^∗∗∗∗^*P* < 0.0001 versus PBS control.
