# Supplementary material for: Asymptomatic Hyperuricemia Is Associated with Achilles Tendon Rupture through Disrupting the Normal Functions of Tendon Stem/Progenitor Cells
Source: Stem Cells Int. 2022 Dec 1;2022:6795573. doi: 10.1155/2022/6795573 (PMC9731760; doi:10.1155/2022/6795573)

Supplementary Table 1

Primer sequences used in real-time PCR.

Gene	Forward (5’→3’)	Reverse (5’→3’)
MMP2	CAACTACAACTTCTTTCCCCGC	TTGATCATGATGTCAGCTTCCC
MMP3	ATCCCCTGATGTCCTCGTGGT	GGGTCCTGAGAGATTTTCGCC
MMP13	CCAACCCTAAGCACCCCAAAAC	TCCTCAAAGTGAACCGCAGCAC
IL1β	GCACCTTCTTTTCCTTCATCTTTG	TTTGTCGTTGCTTGTCTCTCCTT
IL6	TCCTACCCCAACTTCCAATGCTC	TTGGATGGTCTTGGTCCTTAGCC
TNFα	AAATGGGCTCCCTCTCATCAGTTC	CCGCTTGGTGGTTTGCTACGAC
β-Actin	TCAGGTCATCACTATCGGCAAT	AAAGAAAGGGTGTAAAACGCAG

Supplementary Figure 1

Pearson correlations of age and ATR incidence (P < 0.0001) . The incidence of ATR were calculated as the percentile of ATR patient numbers to the total number of participants of the same age. Age range were 18-60 years old, as matched to the control group.

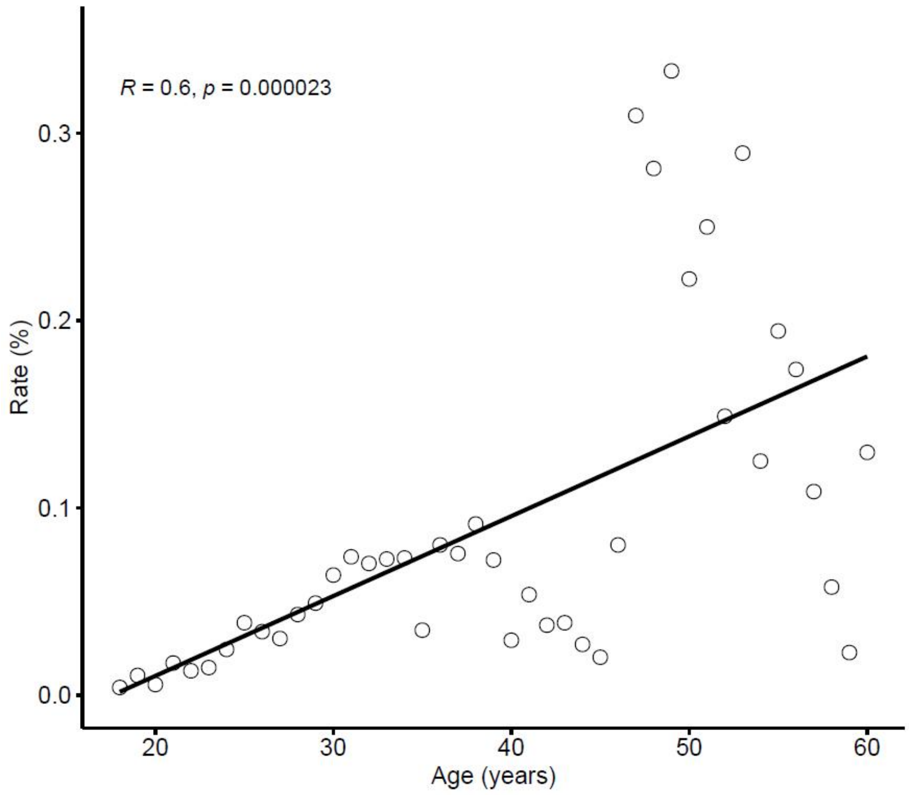

Supplementary Figure 2

Flow cytometric analysis of Annexin V-PI staining results for TSPCs treated with different doses of UA.

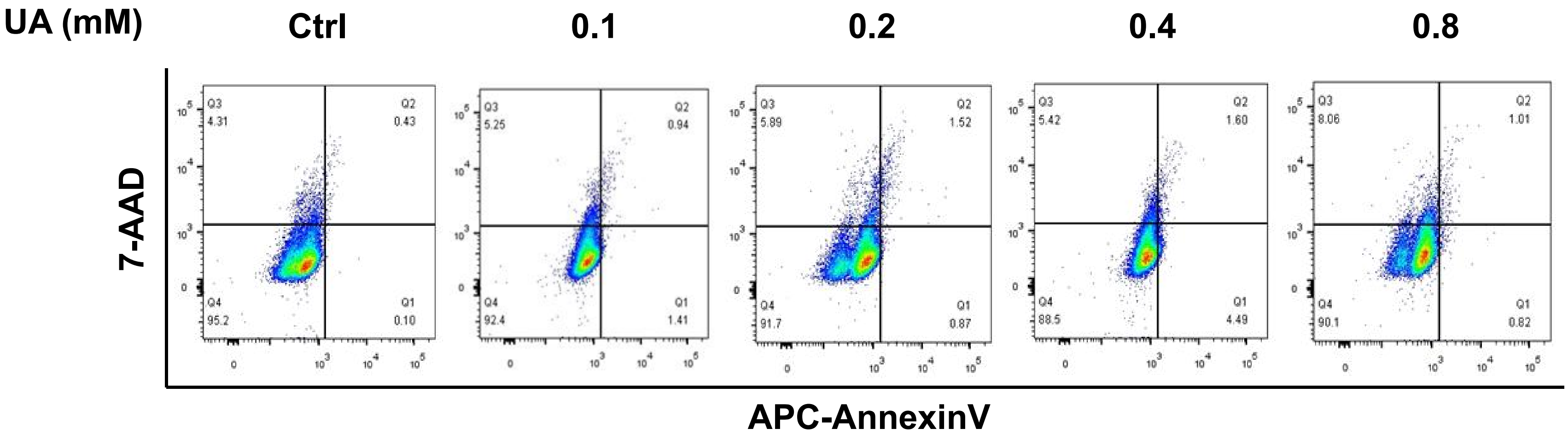

Supplementary Figure 3

Quantification for the Oil Red-O staining (**a**), Alizarin Red staining (**b**) and Safranin-O staining (**c**) staining images in Figure 3 of TSPCs differentiation potential when treated with different doses of UA (n = 3). Data were the normalized ratio of staining intensity of each treatment group over that of the PBS control. Values are the mean ± SD staining intensity of representative images of three independent experiments. *: *P* < 0.05; **: *P* < 0.01; ****: *P* < 0.0001 versus PBS control.

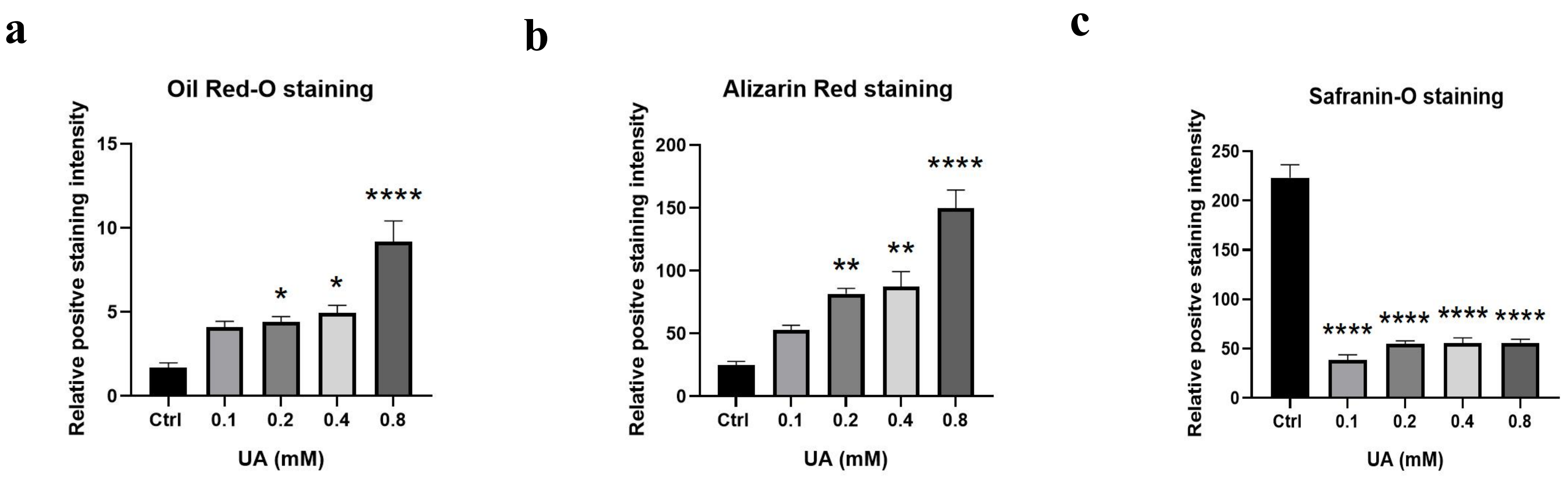

Supplementary Figure 4

Quantification of CD3 (**a**) and CD68 (**b**) immunofluorescent staining results in Figure 4b and 4c, respectively (n = 3). Values are the mean ± SD staining intensity of representative images of three independent experiments. Real-time qPCR results of pro-inflammatory markers (**c**) and degradative enzymes (**d**) in TSPCs treated with different concentrations of UA or PBS (n = 3 each). Values show the mean ± SEM. *: $P < 0.05$; **: $P < 0.01$; ***: $P < 0.001$; ****: $P < 0.0001$ versus PBS-treated control within each group, analyzed using two-way ANOVA. Results are from two independent experiments.

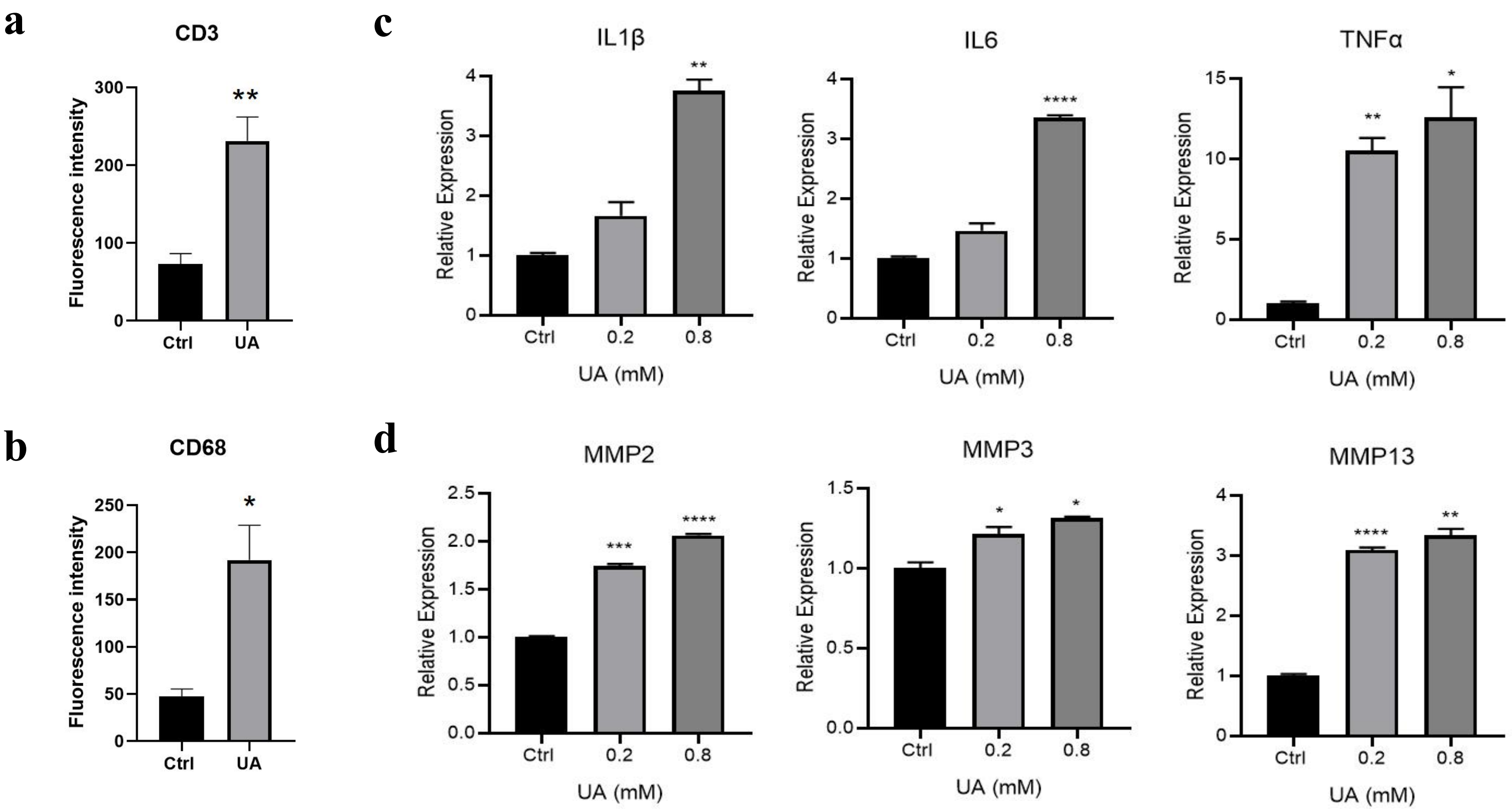

Supplementary Figure 5

Venn diagrams illustrate the sharing of DEG between TSPCs treated with control (PBS) or different concentrations of UA. Ctrl: TSPCs treated with PBS, T02: TSPCs treated with 0.2 mM of UA, T08: TSPCs treated with 0.8 mM UA. The numbers (13, 29, 27 and 3) indicate the DEG between two groups.

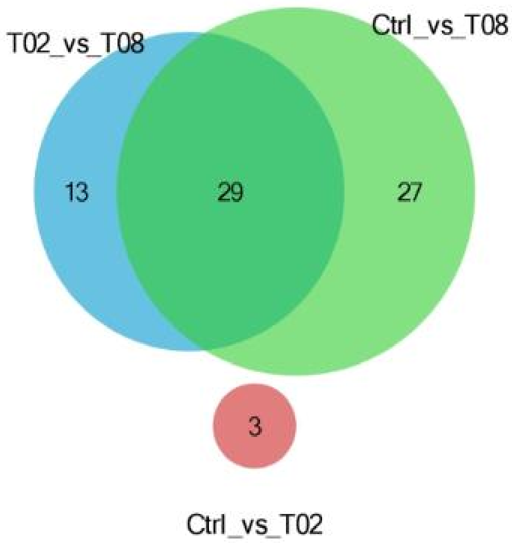

Supplementary Figure 6

Quantification for Western blot analysis images in Figure 5C of TSPCs treated with different doses of UA (n = 3). Data were the normalized ratio of indicated protein expression over that of the corresponding loading control. Values are the mean ± SEM from three independent experiments. *: $P < 0.05$; **: $P < 0.01$; ***: $P < 0.001$; ****: $P < 0.0001$ versus PBS control.

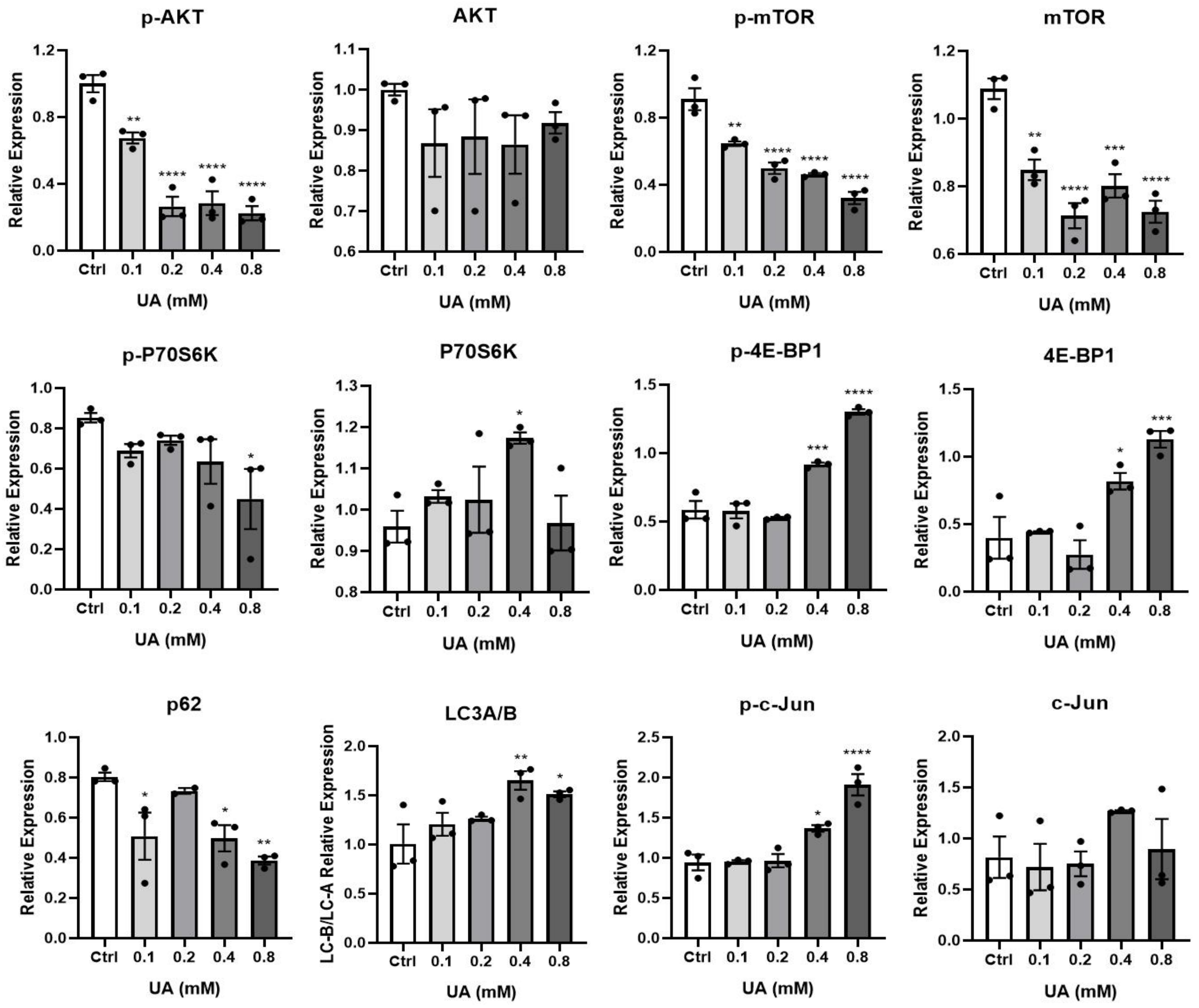

Supplement: Supplementary Materials — Supplementary Table 1: primer sequences used in real-time PCR. Supplementary Figure 1: Pearson's correlations of age and ATR incidence (P < 0.0001). The incidences of ATR were calculated as the percentile of ATR patient numbers to the total number of participants of the same age. Age range was 18-60 years old, as matched to the control group. Supplementary Figure 2: flow cytometric analysis of annexin V-PI staining results for TSPCs treated with different doses of UA. Supplementary Figure 3: quantification for the oil red O staining (a), alizarin red staining (b), and safranin O staining (c) images in Figure 3 of TSPC differentiation potential when treated with different doses of UA (n = 3). Data were the normalized ratio of staining intensity of each treatment group over that of the PBS control. Values are the mean ± SD staining intensity of representative images of three independent experiments. ∗P < 0.05; ∗∗P < 0.01; ∗∗∗∗P < 0.0001 versus PBS control. Supplementary Figure 4: quantification of CD3 (a) and CD68 (b) immunofluorescent staining results in Figures 4(b) and 4(c), respectively (n = 3). Values are the mean ± SD staining intensity of representative images of three independent experiments. Real-time qPCR results of proinflammatory markers (c) and degradative enzymes (d) in TSPCs treated with different concentrations of UA or PBS (n = 3 each). Values show the mean ± SEM. ∗P < 0.05; ∗∗P < 0.01; ∗∗∗P < 0.001; ∗∗∗∗P < 0.0001 versus PBS-treated control within each group analyzed using two-way ANOVA. Results are from two independent experiments. Supplementary Figure 5: Venn diagrams illustrate the sharing of DEG between TSPCs treated with control (PBS) or different concentrations of UA. Ctrl: TSPCs treated with PBS; T02: TSPCs treated with 0.2 mM of UA; T08: TSPCs treated with 0.8 mM UA. The numbers (13, 29, 27, and 3) indicate the DEG between two groups. Supplementary Figure 6: quantification for Western blot analysis images in Figure 5(c) of TSPCs treated with [file 6795573.f1.pdf]
